# High-resolution fish on DNA fibers for low-copy repeats genome architecture studies

**DOI:** 10.1016/j.ygeno.2012.08.007

**Published:** 2012-12

**Authors:** O. Molina, J. Blanco, E. Anton, F. Vidal, E.V. Volpi

**Affiliations:** a Unitat de Biologia Cel·lular, Facultat de Biociències, Universitat Autònoma de Barcelona, 08193 Bellaterra (Cerdanyola del Vallès), Spain; b The Wellcome Trust Center for Cell Biology, University of Edinburgh, Michael Swann Building, King's Buildings, Mayfield Road, Edinburgh EH9 3JR, UK; c The Wellcome Trust Center for Human Genetics, University of Oxford, Roosevelt Drive, Oxford OX3 7BN, UK

**Keywords:** Fiber-FISH, Low copy repeats, Non-allelic homologous recombination

## Abstract

Low-copy repeats (LCRs) constitute 5% of the human genome. LCRs act as substrates for non-allelic homologous recombination (NAHR) leading to genomic structural variation. The aim of this study was to assess the potential of Fiber-FISH for LCRs direct visualization to support investigations of genome architecture within these challenging genomic regions. We describe a set of Fiber-FISH experiments designed for the study of the LCR22-2. This LCR is involved in recurrent reorganizations causing different genomic disorders. Four fosmid clones covering the entire length of the LCR22-2 and two single-copy BAC-clones, delimiting the LCR22-2 proximally and distally, were selected. The probes were hybridized in different multiple color combinations on DNA fibers from two karyotypically normal cell lines. We were able to identify three distinct structural haplotypes characterized by differences in copy-number and arrangement of the LCR22-2 genes and pseudogenes. Our results show that Multicolor Fiber-FISH is a viable methodological approach for the analysis of genome organization within complex LCR regions.

## Highlights

► We describe a Fiber-FISH experiment to analyze the LCR22-2 genomic organization. ► Three different haplotypes were identified in two cell lines. ► Fiber-FISH enables the analysis of the genome architecture of complex LCRs.

## 1 Introduction

It has been estimated that the 5% of the human genome is constituted by segmental duplications or Low Copy Repeats (LCRs) [1]. LCRs are repetitive DNA elements from 1 to 400 Kb in length sharing a high level of sequence identity (> 95%) [2]. Due to the high degree of homology between paralogous copies, they are considered highly dynamic regions leading to genomic instability by non-allelic homologous recombination (NAHR) [3,4].

As a result, LCRs are susceptible to structural and copy-number variation of their own genes and pseudogenes. This variation has been directly associated to the occurrence of some diseases [5–7] and to the formation of specific structural haplotypes, which have been linked to an increased susceptibility to secondary rearrangements of the region flanked by the LCR [8]. Rearrangements may be either somatic, causing sporadic disease in the individual, or in the germ line, leading to an increase in the risk of transmission to the offspring [8].

Some LCR haplotypes have been suggested to predispose to specific chromosomal rearrangements: 1) copy number variation (CNV) within some portions of the LCRs flanking the 7q11.23 region has been linked to the occurrence of deletions of the Williams–Beuren syndrome critical region [9], 2) variation in the copy-number and arrangement of the simple LCRs REPA and REPB at the chromosome region 17p11 is believed to confer different susceptibility to the formation of 17p isochromosomes [10], and 3) CNV of duplicated blocks within the BP1 and BP3 at 16p12.1 has recently been reported to predispose to deletions of this region [11].

Despite the recent advances in copy-number and structural variation detection [12], the repeated nature and often complex organization of LCRs hampers their analysis by standard methodologies such as array comparative genomic hybridization (array-CGH) and single nucleotide polymorphism (SNP) microarrays. The development of next-generation sequencing techniques has been successfully used to analyze the CNV of the whole genome [13], as well as specific LCRs; this is the case of the LCR22 of the 22q11.2 region [14]. PCR-based techniques have also allowed the quantification of the number of repeats shaping specific LCRs (7q11.23; [9]).

Fluorescence *in situ* hybridization (FISH) provides an alternative approach for the analysis of the genomic architecture of LCRs. By enabling the direct visualization of target DNA sequences *in situ*, FISH not only allows copy number assessment, but also facilitates the identification of balanced structural variants such as inversions and translocations. In particular, FISH on stretched DNA fibers (Fiber-FISH) with closely spaced probes has been satisfactory applied in several high-resolution physical mapping studies [15–17] and as a validation technique in CNV studies [18–23]. Moreover, it has also been used to assess the number of paralogous copies of the simple LCRs REPA and REPB on the 17p11.2 region [24].

The pericentromeric area of chromosome 22 contains its own LCRs (LCR22). These LCRs are involved in recurrent reorganizations causing different genomic disorders [25]. Among them, the DiGeorege/Velocardiofacial syndrome (DGS) represents the most common deletion-caused syndrome in humans with an incidence of 1 every 4,000 newborns (OMIM188400) [26]. DGS is mostly caused by 3 Mb hemizygous deletions involving the flanking LCR22-2 and LCR22-4. These LCRs are complex mosaic of genes, pseudogenes and other repetitive elements partially formed by *Alu*-mediated recombination events during primate evolution [27]. The functional genes distributed along the LCR22s, are *USP18*, *BCR*, GGT5 and GGT1. Duplications of these genes and their own pseudogenes during evolution shaped the LCR22s [28].

In this work, we applied Fiber-FISH to determine structural and copy number variants within the LCR22-2. The main objective of the study was to assess the ability of Fiber-FISH – as a high-resolution mapping technique – to resolve the genomic architecture of complex LCRs, and to establish its potential as a methodological approach to assess risk haplotypes for critical regions.

## 2 Results

### 2.1 Clone selection and positioning

**Fig. 1:**
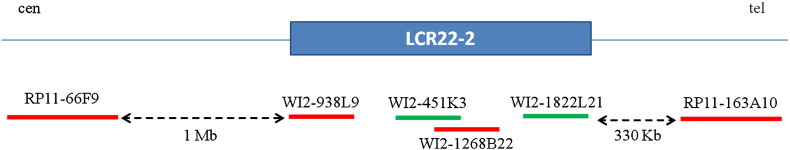
Map of the LCR22-2 (chr22:18,663,074-18,992,962) according to the Genome Browser database (UCSC Assembly Feb 2009) [27]. The length of the LCR is represented as a blue box. The Figure shows the clones used in the study. Bar colors represent the color they were detected with (red: Cy3 and green: FITC) in the two-colour Fiber-FISH experiments.

Four fosmid clones were selected from the Genome Browser database (UCSC Assembly Feb 2009) [29]: WI2-938L9 (abbr. L9), WI2-451K3 (abbr. K3), WI2-1268B22 (abbr. B22) and WI2-1822L21 (abbr. L21). These clones cover total or partially the following UCSC genes and pseudogenes of the LCR22-2: *USP18, AK129567, AK302545, GGT3P, DGCR6* and *PRODH* (Table 1). A single-copy the BAC clone RP11-66F9 (abbr. F9) approximately 1 Mb proximal to the LCR22-2 (UCSC Assembly February 2009) (Fig. 1), a single-copy the BAC clone RP11-163A10 (abbr. A10) approximately 330 Kb distal to the LCR22-2 (UCSC Assembly February 2009) (Fig. 1), and a painting probe for the q arm of chromosome 22 (WCP22) (Cambio) were used as reference probes.

**Fig. 2:**
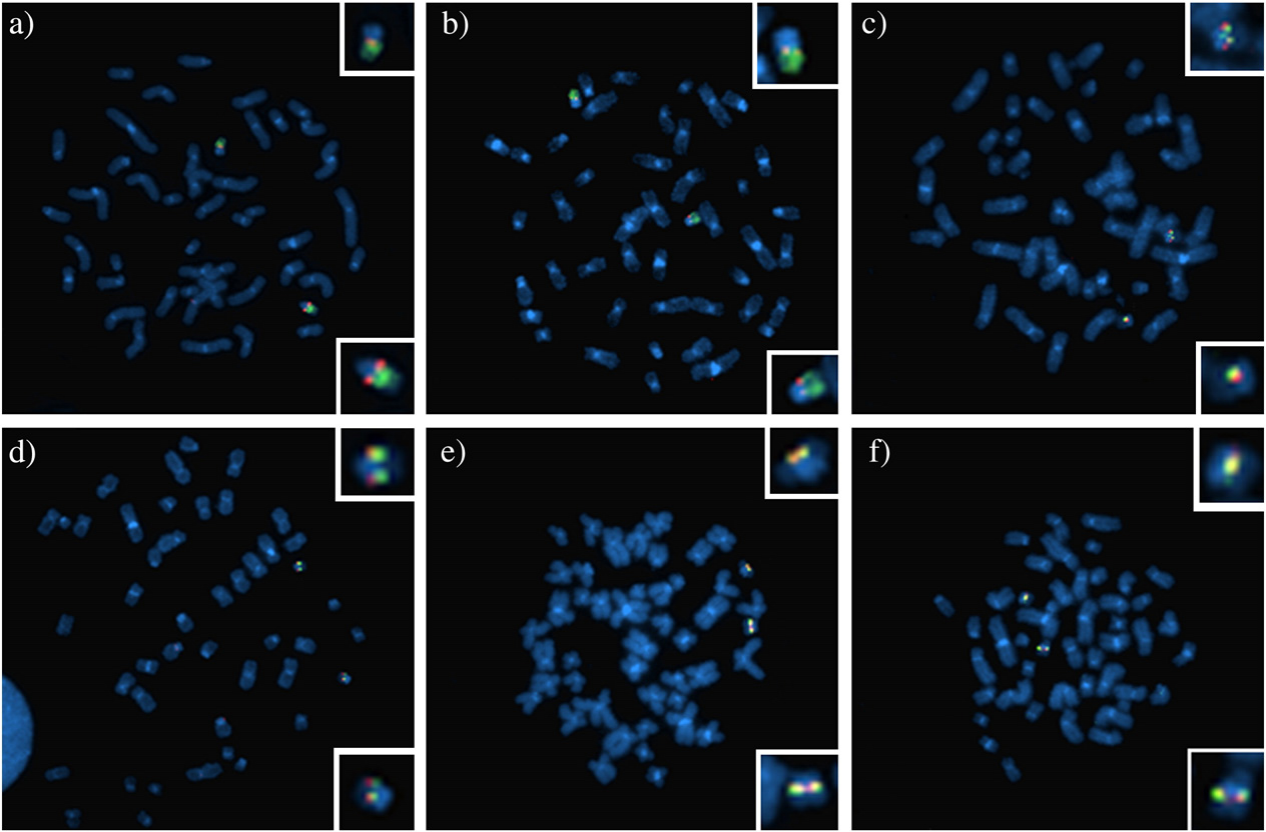
Clone mapping by metaphase FISH a) co-hybridization of WCP22 (green) with F9 (red), b) co-hybridization of WCP22 (green) with A10 (red), c) co-hybridization of F9 (green) with L9 (red), d) co-hybridization of F9 (green) with B22 (red), e) co-hybridization of L9 (green) with K3 (red) and f) co-hybridization of B22 (green) with L21 (red). A closer view of the chromosomes 22 are illustrated in each image.

Chromosomal mapping was verified on metaphase chromosomes using the following combination of probes: F9 and WCP22 (Fig. 2a), A10 and WCP22 (Fig. 2b), F9 and L9 (Fig. 2c), F9 and B22 (Fig. 2d), L9 and K3 (Fig. 2e), B22 and L21 (Fig. 2f).

### 2.2 Experimental design

A set of Fiber-FISH experiments were performed to establish the LCR22-2 genomic architecture in two karyotypically normal cell lines (see section 4: Materials and methods). DNA fibers were stretched on slides as previously described [30]. The experiments were designed as follows:

#### 2.2.1 Unequivocal identification of the specific LCR22-2 signals

To distinguish between specific signals of LCR22-2 from paralogous copies distributed in other LCRs on 22q, the control probes F9 (mapping just outside the LCR22-2, proximally) and A10 (mapping just outside the LCR22-2, distally), were co-hybridized with the fosmid clone K3 in two different FISH experiments, allowing to identify patterns – based on the number of K3 repetitions – to be used as an LCR22-2 reference in the following hybridizations.

#### 2.2.2 Determination of the LCR22-2 architecture

Once the number of K3 copies was established, three dual-color fiber-FISH experiments were performed by co-hybridizing K3 and L9, K3 and B22, B22 and L21. These high-resolution mapping experiments allowed the assessment of the LCR 22‐2 genes copy number and relative arrangement.

### 2.3 Structure of the LCR22-2 in the Cell line A

A total of 81 informative fiber-FISH images were captured and analyzed to study the organization of the LCR22-2 in cell line A.

**Fig. 3:**
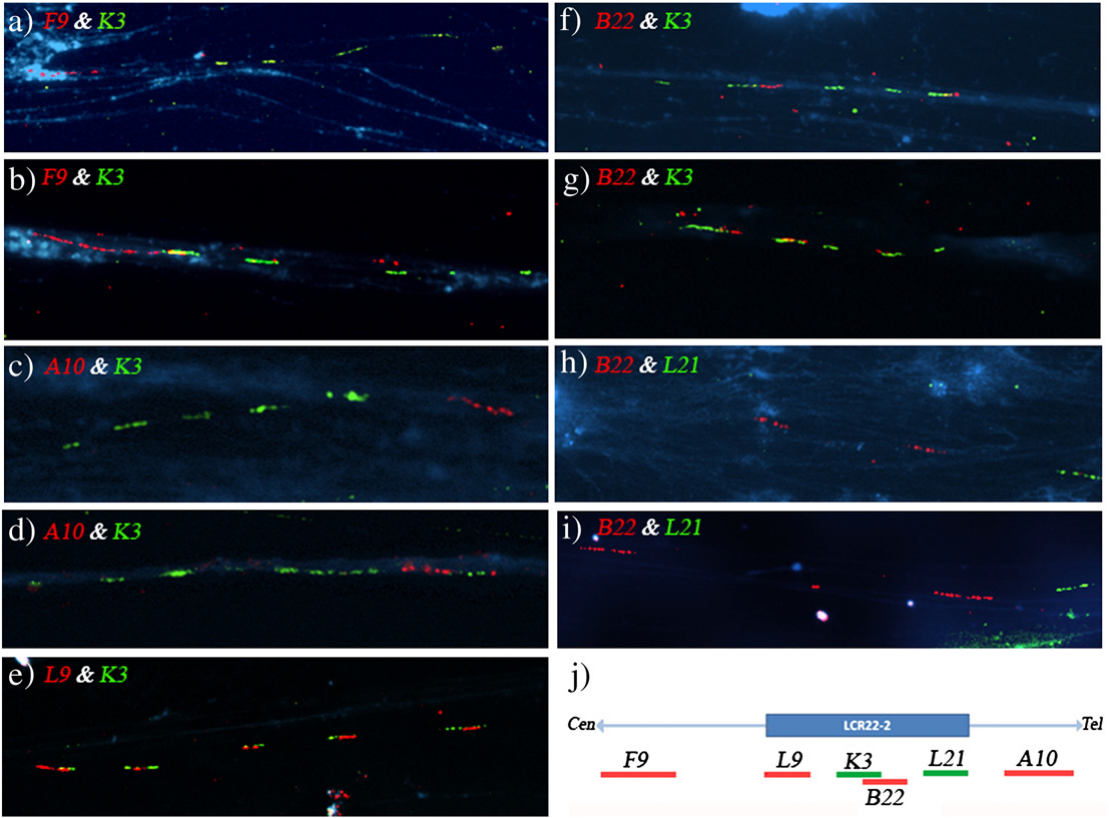
Dual-color Fiber-FISH experiments in the cell line A: a) F9 (red) and K3 (green); b) F9 (red) and K3 (green); c) A10 (red) and K3 (green); d) A10 (red) and K3 (green); e) L9 (red) and K3 (green); f) B22 (red) and K3 (green); g) B22 (red) and K3 (green); h) B22 (red) and L21 (green); i) B22 (red) and L21 (green); j) Diagram showing the positions of the BAC and fosmid probes along the LCR 22‐2.

• F9 and K3: A larger signal corresponding to F9 followed/preceded by a consistent pattern of five repeats for K3 was observed. F9 was either separated (55% of the fibers; Fig. 3a) or overlapping (45% of the fibers; Fig. 3b) with the first/fifth K3 repeat (20 informative fibers were analyzed).
• A10 and K3: A larger signal corresponding to A10 followed/preceded by a consistent pattern of five repeats for K3 was observed. A10 was either separated (62%; Fig. 3c) or overlapping (38%; Fig. 3d) with the first/fifth K3 repeat (13 informative fibers were analyzed).
• K3 and L9: K3 displayed the same pattern previously described. Five signals were identified for L9, totally or partially overlapping with K3 (Fig. 3e) (17 informative fibers were analyzed).
• K3 and B22: Overlapped or partially-overlapped signals from these two probes were observed. Results showed two different patterns for B22 which either two or three signals (52.4% and 47.6% respectively; Figs. 3f and g) (21 informative fibers were analyzed).
• B22 and L21: Two or three signals for B22 (60% and 40% respectively) were observed followed/preceded by one signal for L21 (Figs. 3h and i) (10 informative fibers were analyzed).

**Fig. 4:**
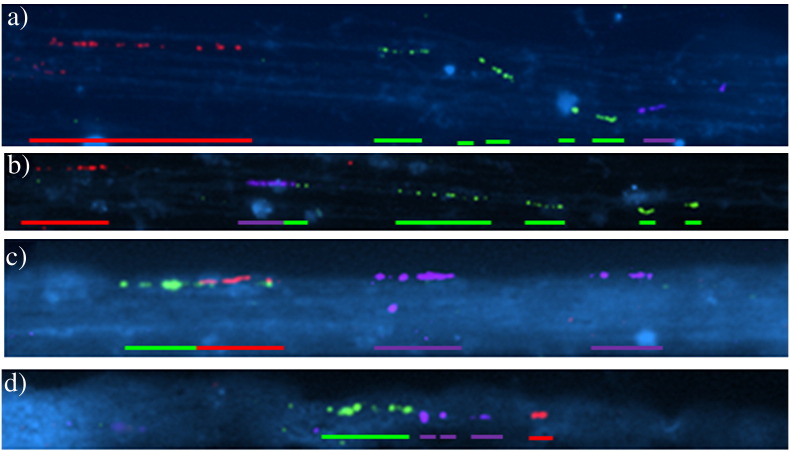
Detection of an inverted haplotype in the cell line A by three-color Fiber-FISH. Color-bars show the signal distributions observed for every single haplotype. a) Co-hybridization of F9 (red), K3 (green) and L21 (purple), signal distribution following the current human genome assembly; b) co-hybridization of F9 (red), K3 (green) and L21 (purple), signal distribution corresponding to an inversion regarding the current human genome assembly; c) co-hybridization of F9 (green), B22 (purple) and L21 (red), signal distribution following the current human genome assembly; d) co-hybridization of F9 (green), B22 (purple) and L21 (red), signal distribution corresponding to an inversion regarding the current human genome assembly.

To determine whether an inversion was the cause of the two different signal patterns or “Fiber-FISH haplotypes” observed co-hybridizing the F9 and K3 clones (Figs. 3a and b) and A10 and K3 (Figs. 3c and d), an additional three-color Fiber-FISH was performed using the probes F9, K3 and L21. Two different signal patterns with the same frequency were observed: 1) F9 followed by K3 and L21 (42.8%; Fig. 4a), and 2) F9 followed by L21 and K3 (57.1%; Fig. 4b). These results strongly suggest the presence of an inversion involving most of the LCR22-2 in one of the two chromosome 22 homologs. In order to relate the number of B22 signals (Figs. 3f and g) with the inversion, a further three-color Fiber-FISH experiment was performed using the clones F9, B22 and L21. Two clone distributions were observed: 1) F9, L21 and two signals of B22 (38%; Fig. 4c), and 2) F9, B22 (three signals) and L21 (62%; Fig. 4d). These results suggest that the inversion segregates with the haplotype showing two signals for B22.

### 2.4 Structure of the LCR22-2 in the cell line B

A total of 87 informative images were analyzed using the following two-color Fiber-FISH experiments.

**Fig. 5:**
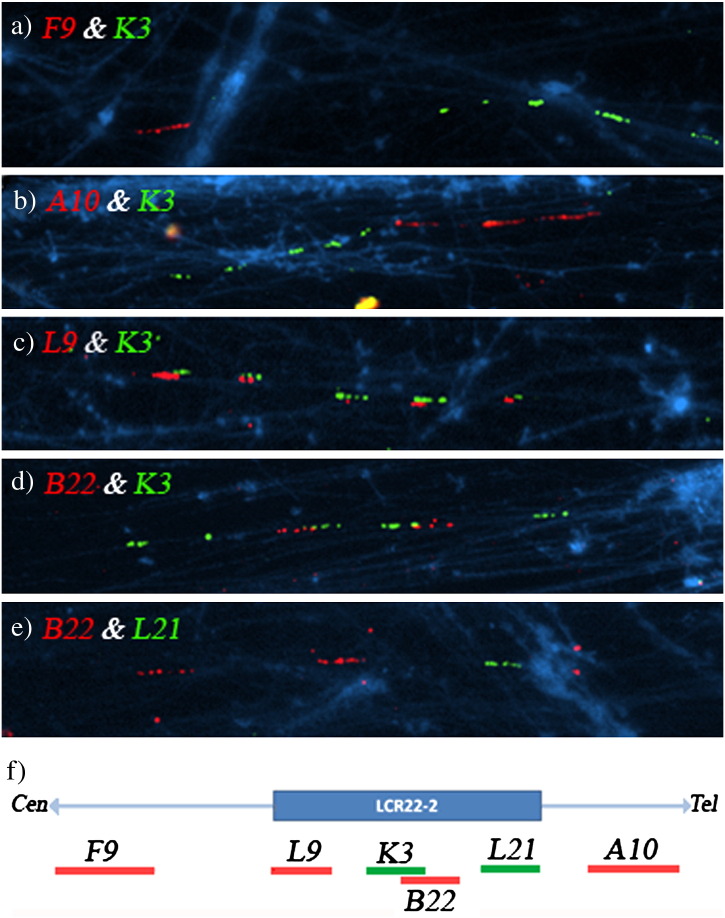
Dual-color Fiber FISH experiments in cell line B: a) F9 (red) and K3 (green); b) A10 (red) and K3 (green); c) L9 (red) and K3 (green); d) B22 (red) and K3 (green); e) B22 (red) and L21 (green); f) Diagram showing the positions of the BAC and fosmid probes along the LCR 22‐2.

• F9 and K3: As observed in cell line A, five contiguous signals for K3 were observed, all of them separated to the F9 signal (Fig. 5a) (18 informative fibers were analyzed).
• A10 and K3: As in cell line A, five contiguous signals for K3 were observed, all of them separated to a longer A10 signal (Fig. 5b) (18 informative fibers were analyzed).
• K3 and L9: As in cell line A, five contiguous signals for K3 and 5 partially overlapped L9 signals were detected (Fig. 5c) (20 informative fibers were analyzed).
• K3 and B22: Two signals for B22 were consistently found on the second and third/third and fourth K3 signals (Fig. 5d) (14 informative fibers were analyzed).
• B22 and L21: Two signals for B22 were observed followed/preceded by one signal for L21 (Fig. 5e) (17 informative fibers were analyzed).

**Fig. 6:**
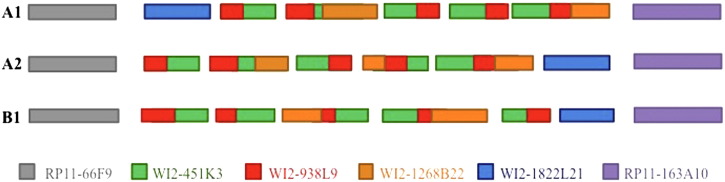
Proposed model for the genomic architecture of the LCR22-2 in the two cell lines analyzed. The two “Fiber-FISH haplotypes” detected in the cell line A were named A1 and A2, and the haplotype detected in the cell line B was names B1.

Results allowed us to propose a model for the architecture of the LCR22-2 in the cell line A and B (Fig. 6).

## 3 Discussion

This work demonstrates for the first time the ability of Fiber-FISH coupled to an accurate experimental design to resolve the genomic architecture of complex LCRs. The strategy used in our study allows the identification of structural variation of different segments within the LCRs. The design consists in: 1) selecting specific clones covering the LCR, 2) selecting chromosomal markers to be used as positional references to facilitate the unequivocal identification of the LCR under investigation, and 3) developing and applying strict assessment criteria for the analysis of the Fiber-FISH hybridization patterns.

By direct visualization of haplotypic repeat patterns, Fiber-FISH allows both inter-chromosomal and inter-individual variability to be reliably ascertained, as our results on the LCR22-2 show (Fig. 6). Our observations suggest an arrangement of the LCR22-2 comprising five copies of the L9 and the K3, and of either two or three copies of the B22, all of them closely localized to each other and repeated in a modular fashion (Fig. 6). Furthermore, we observed an inverted haplotype. The inversion involves most of the LCR22-2; accordingly, the relative position of the L21 was close to the clone F9. Besides, the combination in a triple-color fashion of the probes: 1) F9, K3 and L21, and 2) F9, B22 and L21 confirm again that the signals analyzed unequivocally identify the LCR22-2.

Some LCR structural haplotypes have been suggested to increase the likelihood of misalignment and NAHR, thus increasing the risk of transmission of secondary disease-associated rearrangements to the offspring [9–11]. Moreover, some data demonstrated a different susceptibility to NAHR among individuals. Our group have recently reported increased rates of deletions of the 15q11–q13 region in spermatozoa of fathers of children affected by Prader–Willi syndrome [31], as well as increased rates of 7q11.23 and 22q11.2 deletions in spermatozoa of fathers of Williams–Beuren or DiGeorge/Velocardiofacial children respectively (unpublished data), thus suggesting the presence of predisposing haplotypes to NAHR in these subjects.

The results obtained in this work demonstrate the potential of the Fiber-FISH methodology for the identification of predisposing LCR haplotypes in the flanking critical regions in parents of individuals affected by genomic disorders. This would allow the establishment of a direct relationship between specific LCR structural haplotypes and increased rates of NAHR in gametes.

### 3.1 Conclusion

Multicolor Fiber-FISH is a viable methodological approach for the analysis of genome organization within complex LCR regions.

## 4 Materials and methods

### 4.1 Cell culture

Two karyotypically normal B-lymphoblastoid cell lines were used: 1) GM0171 (Human Genetics Collection, Health Protection Agency (HPA), U.K.; no longer available) and 2) DO208915 (European collection of Cell Cultures, HPA), referred in the manuscript as cell line A and B respectively. Both cell lines harbor the VCFS/DiGeorge region since they showed a normal hybridization pattern with A10 (a clone localized within the critical region).

Cell cultures were grown in RPMI-1640 medium (Sigma-Aldrich), supplemented with 10% fetal bovine serum (Sigma-Aldrich) and 1% l-glutamine at 37 °C in a 5% CO_2_ incubator for 72 hours.

### 4.2 Slide preparation

Two kinds of preparations were performed:

4.2.1) Metaphase chromosomes were obtained following standard procedures: 1 hour before harvesting, cells were treated with Colcemid (Invitrogen) at a final concentration of 0.2 μg/mL. They were then resuspended in hypotonic solution (0.075 M KCl) for 10 minutes at 37 °C and fixed in methanol:acetic acid (3:1).
4.2.2) DNA fibers were stretched on slides as previously described [30]. Briefly, 2 mL of a cell culture were centrifuged and the pellets were washed in 1 × PBS. Pellets were resuspended in 1 × PBS to reach a final concentration of 2 × 10^6^ cells/mL and spread on slides. Once the slides were mounted on the Shandon Sequenza Coverplates DNA fibers were released applying a lysis solution (0.07 M NaOH in ethanol). Finally, fibers were fixed in methanol.

In both cases, slides were kept at − 20 °C until processed.

### 4.3 Probes

All clones were kindly provided by the Wellcome Trust Sanger Institute (Cambridge, UK). Clone extraction was carried out using the QuickClean 5M Miniprep kit (GenScript) following the manufacturer's instructions.

### 4.4 Fluorescence *in situ* hybridization (FISH)

In two-color FISH experiments, clones were labeled by Nick-Translation (Abbott Molecular) either with Digoxigenin-11-dUTP (Roche) or Biotin-16-dUTP (Roche) (Table 1). In three-color experiments, Alexa594-dUTP (Invitrogen) was also used. Probes were ethanol precipitated with a mix of salmon testis DNA (GIBCO-BRL), *Escherichia coli* tRNA (Boehringer) and 3 M sodium acetate. Approximately 200 ng of labeled DNA probe and 4 μg of Cot1 competitor DNA (Invitrogen) were mixed and dried on a heating block at 60 °C, and resuspended in 1 × hybridization buffer (50% formamide, 1 × SSC and 10% dextran sulfate) to a final concentration of 40 ng/μL. For Fiber-FISH experiments, two-fold the labeled DNA probe and Cot1 competitor DNA were used (final concentration of 80 ng/μL of each probe).

FISH was carried out following standard procedures [30]. Briefly, probes were denatured at 75 °C for 5 minutes and pre-annealed at 37 °C for 45 minutes. Slides were denatured in 70% formamide/2 × SSC at 70 °C for 1 minute and hybridized in a moist chamber at 37 °C overnight. Slides were washed twice in 50% formamide/1 × SSC and once in 2 × SSC, for 5 minutes at 42 °C, followed by 5 minutes in 1 × PBS at room temperature. For fiber-FISH experiments milder washes were used: one wash in 50% formamide/1 × SSC, followed by one wash in 2 × SSC, both of them for 5 minutes at 42 °C.

In two-color FISH, probes were detected with either fluorescein-conjugated antidigoxigenin (Roche) or Cy3-conjugated Streptavidin (Sigma). In the three-color experiments, Alexa594 directly labeled-probes were used together with probes labeled with digoxigenin and biotin that were detected by fluorescein-conjugated antidigoxigenin (Roche) and Cy5-conjugated Streptavidin (CyDye, Amersham Pharmacia Biotech) respectively. The slides were mounted with Vectashield (Vector Laboratories) containing 4′, 6-diamidino-2-phenylindole (DAPI) for chromosome counterstaining.

### 4.5 Image acquisition and data analyses

Image capture and analysis were carried out on a CytoVision system (Leica) consisting of an Olympus BX-51 epifluorescence microscope coupled to a JAI CVM4 + CCD camera.

Fiber-FISH analysis was performed by applying the following scoring criteria:

• Fibers were considered informative when at least two signals of different colors were observed overlapping or proximal in a consecutive fashion.
• Two or more signals of the same color were considered independent when they were separated by a distance twice the distance of every single bead-on-string.
• Signals were considered informative regardless of the size.

## Author's contributions

O.M. was responsible for conception and design, acquisition of data, data analysis and interpretation, writing the article and final approval. J.B. was responsible for conception and design, data analysis and interpretation, writing the article and final approval. E.A. was responsible for revision of data and interpretation and final approval. F.V. was responsible for revision of data and interpretation and final approval. E.V.V. was responsible for conception and design, data analysis and interpretation, and final approval.

## Figures and Tables

**Table 1:** Characteristics of the clones (UCSC Assembly Feb 2009) [27].

Clone	Whitehead F.E.S. name	Size (Kb)	Position	UCSC genes	Labeling
WI2-938L9	G248P80545F5	37	chr22:18,631,074-18,672,626	*USP18* *AK129567*^a^	Biotin
WI2-451K3	G248P8704F2	44	chr22:18,725,757-18,770,041	*AK302545*^a^ *GGT3P*	Digoxigenin
WI2-1268B22	G248P82259A11	40	chr22:18,751,182-18,793,055	*GGT3P*	Biotin
WI2-1822L21	G248P86641F11	39	chr22:18,883,978-18,922,962	*DGCR6* *PRODH*^a^	Digoxigenin
RP11-66F9	–	175	chr22:17,381,569-17,556,908	*GAP 4* *CECR7*	Biotin
RP11-163A10	–	183	chr22:19,323,802-19,506,889	*HIRA*	Biotin

a Partially covered genes.

## References

[1] She X., Jiang Z., Clark R.A., Liu G., Cheng Z., Tuzun E., Church D.M., Sutton G., Halpern A.L., Eichler E.E. (2004). Shotgun sequence assembly and recent segmental duplications within the human genome. Nature.

[2] Emanuel B.S., Shaikh T.H. (2001). Segmental duplications: an ‘expanding’ role in genomic instability and disease. Nat. Rev. Genet..

[3] Lupski J.R. (1998). Genomic disorders: structural features of the genome can lead to DNA rearrangements and human disease traits. Trends Genet..

[4] Stankiewicz P., Lupski J.R. (2010). Structural variation in the human genome and its role in disease. Annu. Rev. Med..

[5] Gonzalez E., Kulkarni H., Bolivar H., Mangano A., Sanchez R., Catano G., Nibbs R.J., Freedman B.I., Quinones M.P., Bamshad M.J., Murthy K.K., Rovin B.H., Bradley W., Clark R.A., Anderson S.A., O'Connell R J., Agan B.K., Ahuja S.S., Bologna R., Sen L., Dolan M.J., Ahuja S.K. (2005). The influence of CCL3L1 gene-containing segmental duplications on HIV-1/AIDS susceptibility. Science.

[6] Aitman T.J., Dong R., Vyse T.J., Norsworthy P.J., Johnson M.D., Smith J., Mangion J., Roberton-Lowe C., Marshall A.J., Petretto E., Hodges M.D., Bhangal G., Patel S.G., Sheehan-Rooney K., Duda M., Cook P.R., Evans D.J., Domin J., Flint J., Boyle J.J., Pusey C.D., Cook H.T. (2006). Copy number polymorphism in Fcgr3 predisposes to glomerulonephritis in rats and humans. Nature.

[7] Fellermann K., Stange D.E., Schaeffeler E., Schmalzl H., Wehkamp J., Bevins C.L., Reinisch W., Teml A., Schwab M., Lichter P., Radlwimmer B., Stange E.F. (2006). A chromosome 8 gene-cluster polymorphism with low human beta-defensin 2 gene copy number predisposes to Crohn disease of the colon. Am. J. Hum. Genet..

[8] Zhang F., Gu W., Hurles M.E., Lupski J.R. (2009). Copy number variation in human health, disease, and evolution. Annu. Rev. Genomics Hum. Genet..

[9] Cusco I., Corominas R., Bayes M., Flores R., Rivera-Brugues N., Campuzano V., Perez-Jurado L.A. (2008). Copy number variation at the 7q11.23 segmental duplications is a susceptibility factor for the Williams–Beuren syndrome deletion. Genome Res..

[10] Carvalho C.M., Lupski J.R. (2008). Copy number variation at the breakpoint region of isochromosome 17q. Genome Res..

[11] Antonacci F., Kidd J.M., Marques-Bonet T., Teague B., Ventura M., Girirajan S., Alkan C., Campbell C.D., Vives L., Malig M., Rosenfeld J.A., Ballif B.C., Shaffer L.G., Graves T.A., Wilson R.K., Schwartz D.C., Eichler E.E. (2010). A large and complex structural polymorphism at 16p12.1 underlies microdeletion disease risk. Nat. Genet..

[12] Aten E., White S.J., Kalf M.E., Vossen R.H., Thygesen H.H., Ruivenkamp C.A., Kriek M., Breuning M.H., den Dunnen J.T. (2008). Methods to detect CNVs in the human genome. Cytogenet. Genome Res..

[13] Alkan C., Kidd J.M., Marques-Bonet T., Aksay G., Antonacci F., Hormozdiari F., Kitzman J.O., Baker C., Malig M., Mutlu O., Sahinalp S.C., Gibbs R.A., Eichler E.E. (2009). Personalized copy number and segmental duplication maps using next-generation sequencing. Nat. Genet..

[14] Guo X., Freyer L., Morrow B., Zheng D. (2011). Characterization of the past and current duplication activities in the human 22q11.2 region. BMC Genomics.

[15] Raap A.K., Florijn R.J., Blonden L.A.J., Wiegant J., Vaandrager J.W., Vrolijk H., den Dunnen J., Tanke H.J., van Ommen G.J. (1996). Fiber FISH as a DNA mapping tool. Methods.

[16] Protopopov A., Kashuba V., Zabarovska V.I., Muravenko O.V., Lerman M.I., Klein G., Zabarovsky E.R. (2003). An integrated physical and gene map of the 3.5-Mb chromosome 3p21.3 (AP20) region implicated in major human epithelial malignancies. Cancer Res..

[17] Leipoldt M., Erdel M., Bien-Willner G.A., Smyk M., Theurl M., Yatsenko S.A., Lupski J.R., Lane A.H., Shanske A.L., Stankiewicz P., Scherer G. (2007). Two novel translocation breakpoints upstream of SOX9 define borders of the proximal and distal breakpoint cluster region in campomelic dysplasia. Clin. Genet..

[18] Erdel M., Hubalek M., Lingenhel A., Kofler K., Duba H.C., Utermann G. (1999). Counting the repetitive kringle-IV repeats in the gene encoding human apolipoprotein(a) by fibre-FISH. Nat. Genet..

[19] Sallinen R., Vihola A., Bachinski L.L., Huoponen K., Haapasalo H., Hackman P., Zhang S., Sirito M., Kalimo H., Meola G., Horelli-Kuitunen N., Wessman M., Krahe R., Udd B. (2004). New methods for molecular diagnosis and demonstration of the (CCTG)n mutation in myotonic dystrophy type 2 (DM2). Neuromuscul. Disord..

[20] Iafrate A.J., Feuk L., Rivera M.N., Listewnik M.L., Donahoe P.K., Qi Y., Scherer S.W., Lee C. (2004). Detection of large-scale variation in the human genome. Nat. Genet..

[21] Perry G.H., Dominy N.J., Claw K.G., Lee A.S., Fiegler H., Redon R., Werner J., Villanea F.A., Mountain J.L., Misra R., Carter N.P., Lee C., Stone A.C. (2007). Diet and the evolution of human amylase gene copy number variation. Nat. Genet..

[22] Perry G.H., Yang F., Marques-Bonet T., Murphy C., Fitzgerald T., Lee A.S., Hyland C., Stone A.C., Hurles M.E., Tyler-Smith C., Eichler E.E., Carter N.P., Lee C., Redon R. (2008). Copy number variation and evolution in humans and chimpanzees. Genome Res..

[23] Shimojima K., Imai K., Yamamoto T. (2010). A de novo 22q11.22q11.23 interchromosomal tandem duplication in a boy with developmental delay, hyperactivity, and epilepsy. Am. J. Med. Genet. A..

[24] Barbouti A., Stankiewicz P., Nusbaum C., Cuomo C., Cook A., Hoglund M., Johansson B., Hagemeijer A., Park S.S., Mitelman F., Lupski J.R., Fioretos T. (2004). The breakpoint region of the most common isochromosome, i(17q), in human neoplasia is characterized by a complex genomic architecture with large, palindromic, low-copy repeats. Am. J. Hum. Genet..

[25] Emanuel B.S. (2008). Molecular mechanisms and diagnosis of chromosome 22q11.2 rearrangements. Dev. Disabil. Res. Rev..

[26] OMIM Online Mendelian Inheritance in Man. http://www.ncbi.nlm.nih.gov/omim.

[27] Babcock M., Pavlicek A., Spiteri E., Kashork C.D., Ioshikhes I., Shaffer L.G., Jurka J., Morrow B.E. (2003). Shuffling of genes within low-copy repeats on 22q11 (LCR22) by Alu-mediated recombination events during evolution. Genome Res..

[28] Babcock M., Yatsenko S., Hopkins J., Brenton M., Cao Q., de Jong P., Stankiewicz P., Lupski J.R., Sikela J.M., Morrow B.E. (2007). Hominoid lineage specific amplification of low-copy repeats on 22q11.2 (LCR22s) associated with velo-cardio-facial/digeorge syndrome. Hum. Mol. Genet..

[29] UCSC UCSC Genome Bioinformatics. http://genome.ucsc.edu/.

[30] Jefferson A., Volpi E.V. (2010). Fluorescence in situ hybridization (FISH) for genomic investigations in rat. Methods Mol. Biol..

[31] Molina O., Blanco J., Vidal F. (2010). Deletions and duplications of the 15q11–q13 region in spermatozoa from Prader–Willi syndrome fathers. Mol. Hum. Reprod..

